# Symplectic QSD, LCD, and ACD Codes over a Non-Commutative Non-Unitary Ring of Order Nine

**DOI:** 10.3390/e27090973

**Published:** 2025-09-18

**Authors:** Sarra Manseri, Patrick Solé, Adel Alahmadi, Widyan Basaffar

**Affiliations:** 1 Department of Mathematics, Central China Normal University, Wuhan 430079, China; sarramanseri@gmail.com; 2 I2M, (CNRS, University of Aix-Marseille, Centrale Marseille), 13009 Marseilles, France; 3 Research Group of Algebraic Structures and Applications, Mathematics Department, King Abdulaziz University, Jeddah 22254, Saudi Arabia; whbasaffar@kau.edu.sa

**Keywords:** ternary codes, non-unitary ring, symplectic QSD codes, LCD codes, ACD codes, 94B05, 13H99

## Abstract

We introduce quasi self-dual (QSD), linear complementary dual (LCD), and additive complementary dual (ACD) codes for the symplectic inner product over a non-commutative non-unitary ring of order 9. We establish connections with symplectic–self-orthogonal and LCD ternary codes. We characterize right-symplectic ACD codes.

## 1. Introduction

Codes over non-unitary rings have received significant attention in the last five years [1,2] especially in the context of duality [3,4,5,6]. The notion of quasi self-dual codes (QSD) plays here the role of self-dual codes over unitary rings and finite fields.

Recently, Alahmadi et al. [7] derived a mass formula for self-orthogonal, QSD and self-dual codes of non-unitary rings of four elements. Later, this research was extended to non-unitary rings of the size of the square of an odd prime [5,6]. Furthermore, in [3], the concept of linear complementary dual (LCD) codes was characterized over a non-unitary ring of order four. Additive complementary dual (ACD) codes were introduced in [4]. Kushwaha et al. [8] pushed further, with a study in the characteristic three. These studies were conducted using the Euclidean inner product.

More recently, in a series of papers [9,10,11], we introduced and characterized self-orthogonal and QSD codes over non-unitary rings of the order of four under the symplectic inner product. Later, we classified all distinct symplectic–self-orthogonal and QSD codes over commutative non-unitary rings with four elements [9,11]. Additionally, [10] introduced symplectic LCD and ACD codes over a non-commutative non-unitary ring of size four, in which the authors focused on the study of symplectic left ACD and LCD codes. This motivated us to extend the study of left- and right-symplectic LCD and ACD codes to the non-commutative non-unitary ring of order nine. Therefore, in the present manuscript, we consider the case of a ring of order 9 and *E* in the notation of Fine [12]. By forgetting the module structure, our codes can be considered as additive codes over $F_{9},$ a viewpoint that allows us, for numerical examples, to use the additive codes Magma package [13]. Moreover, we introduce QSD, right (respectively, left) ACD, nice, and LCD codes for the symplectic inner product over that ring, here denoted by $E_{3}$. We establish connections with ternary symplectic–self-orthogonal and symplectic LCD codes. In particular, we use a multilevel construction from ternary symplectic–self-orthogonal codes to construct symplectic QSD codes. We investigate the right-symplectic LCD codes. Furthermore, we characterize free right-symplectic LCD codes over $E_{3}$ in terms of a ternary generator matrix. Since non-trivial left-symplectic ACD codes do not exist, we introduce the concept of a right-symplectic ACD code over $E_{3}$. Additionally, we establish several results for the existence of these codes.

This manuscript is organized as follows: The basic notions and notations used in the remainder of this paper are presented in the next section. Section 3 studies the symplectic QSD and LCD codes over $E_{3}$. Section 4 treats right-symplectic ACD $E_{3}$ codes. Section 5 provides the conclusion.

## 2. Preliminaries

### 2.1. Ternary Linear Codes

A linear code *C* having a dimension, *k*, and length, *n*, over the ternary field $F_{3}$ is an $F_{3}$ subspace of $F_{3}^{n}$ of the *k* dimension. Briefly, we say *C* is an [n,k]–code and its elements are known by codewords.

Denote the vector $\left(x_{1}|x_{2}\right)$ as the juxtaposition of two vectors, $x_{1},x_{2}\in F_{3}^{2n}$. Let $x=(x_{1}|x_{2})$ and $y=(y_{1}|y_{2})$, where $x_{1}=\left(x_{1},x_{2},\cdots ,x_{n}\right),\;x_{2}=\left(x_{n+1},x_{n+2},\cdots ,x_{2n}\right)\in F_{3}^{2n},\;y_{1}=\left(y_{1},y_{2},\cdots ,y_{n}\right),\;y_{2}=\left(y_{n+1},y_{n+2},\cdots ,y_{2n}\right)\in F_{3}^{2n}$ are the vectors of $F_{3}^{2n}$. The Euclidean and symplectic inner products of x and y are, respectively, defined by

$${\left(x,y\right)}_{e}=\sum_{i=1}^{2n}x_{i}y_{i}\mathrm{and}{\left(x,y\right)}_{s}={\left(x,y\Omega_{n}^{T}\right)}_{e}={\left(x_{1},y_{2}\right)}_{e}-{\left(x_{2},y_{1}\right)}_{e}=\sum_{i=1}^{n}\left(x_{i}y_{n+i}-x_{n+i}y_{i}\right),$$

where $\Omega_{n}=\left[\begin{array}{cc} 0_{n} & I_{n}\\ -I_{n} & 0_{n} \end{array}\right]$ with $0_{n}$ is the n×n zero matrix and $I_{n}$ is the identity matrix.

Two vectors x and y are symplectic–orthogonals if they satisfy ${\left(x,y\right)}_{s}=0$. Moreover, the vector space consists of all vectors of $F_{3}^{2n}$ that are symplectic–orthogonal to each codeword in *C*, which is known by the symplectic dual of *C*, denoted by $C^{{⊥}_{s}}$.

The code *C* is said to be **symplectic–self-orthogonal** (resp. **symplectic self-dual**) if $C\subseteq C^{{⊥}_{s}}$ (resp. $C=C^{{⊥}_{s}}$).

### 2.2. Non-Commutative Non-Unitary Ring of Nine Elements

Define the ring $E_{3}$ on two generators, *a* and *b*, under certain relations, such as:

$$E_{3}=\langle a,b|\;3a=3b=0,ab=a,ba=b,a^{2}=a,b^{2}=b\rangle .$$

Assume that c=a+2b. According to [8], the ring $E_{3}$ has characteristic 3 and 9 elements, given by

$$E_{3}=\langle 0,a,b,c,2a,2b,2c,a+b,a+c\rangle .$$

It is easy to derive the addition table of $E_{3}$ and its multiplication table is in Table 1.

Thus, $E_{3}$ is a non-commutative ring without identity element with respect to the given multiplication and has a unique maximal ideal J={0,c,2c}. So, the ring $E_{3}$ is local having a residue class field $E_{3}/J≅F_{3}$.

Every element, $r\in E_{3}$, has a *c*-adic decomposition, expressed as r=au+cv, for unique scalars u,v of $F_{3}$. Furthermore, ax=x, bx=x, and cx=0 for all $x\in E_{3}$.

Define a natural action of $F_{3}$ on the ring $E_{3}$ as

r0=0r=0,r1=1r=r,andr2=2r=2r.

This action is clearly distributive in the sense that, for any $r\in E_{3}$ and $u,vs.\in F_{3}$, we have r(u⊕v)=ru+rv, where ⊕ is denoted in the addition in $F_{3}$.

The symplectic inner product of the two elements $u\in F_{3}^{2n}$ and $r\in E_{3}^{2n}$, denoted by ${\left(u,r\right)}_{s}$ is defined as

$${\left(u,r\right)}_{s}=\sum_{i=1}^{n}\left(u_{i}r_{n+i}-u_{n+i}r_{i}\right).$$

We define the reduction map modulo J as: $\alpha_{E_{3}}:E_{3}\to E_{3}/J≅F_{3}$ by

$$\begin{array}{c} \alpha_{E_{3}}\left(0\right)=\alpha_{E_{3}}\left(c\right)=\alpha_{E_{3}}\left(2c\right)=0,\\ \alpha_{E_{3}}\left(a\right)=\alpha_{E_{3}}\left(b\right)=\alpha_{E_{3}}\left(a+c\right)=1,\\ \alpha_{E_{3}}\left(2a\right)=\alpha_{E_{3}}\left(2b\right)=\alpha_{E_{3}}\left(a+b\right)=2. \end{array}$$

This map can be extended naturally to a map from $E_{3}^{n}$ to $F_{3}^{n}$.

### 2.3. Linear $E_{3}$ Codes

A linear code *C* over $E_{3}$ or a linear $E_{3}$ –code of length *n* is a right $E_{3}$ submodule of $E_{3}^{n}$. A linear $E_{3}$ –code *C* is free if it is free as a right $E_{3}$–module.

An additive code over $E_{3}$ or an additive $E_{3}$–code of length *n* is an additive subgroup of $E_{3}^{n}$.

For any linear code *C* over $E_{3}$, there are the two ternary codes of length *n* associated with *C*.

The **residue** code of *C* is defined as
  $$\mathrm{res}\left(C\right)=\{\alpha_{E_{3}}\left(r\right)|\;r\in C\}.$$

The **torsion** code of *C* is

$$\mathrm{tor}\left(C\right)=\left\{u\in F_{3}^{n}|\;uc\in C\right\}.$$

From [2], we have res(C)⊆tor(C). We denote $k_{1}$ as the dimension of res(C), and $k_{1}+k_{2}$ as the dimension of tor(C). We say that a linear $E_{3}$ code, *C*, is of *type* $\left\{k_{1},k_{2}\right\}$ and $\left|C\right|=\left|\mathrm{res}\left(C\right)||\mathrm{tor}\left(C\right)\right|=3^{2k_{1}+k_{2}}$. From [8], *C* is free if, and only if, $k_{2}=0$.

The symplectic inner product between two codewords, $x=\left(x_{1},x_{2},\cdots ,x_{2n}\right),y=\left(y_{1},y_{2},\cdots ,y_{2n}\right)\in C$, is defined as

$${\left(x,y\right)}_{s}=\sum_{i=1}^{n}\left(x_{i}y_{n+i}-x_{n+i}y_{i}\right).$$

Under this symplectic inner product, the duals of an $E_{3}$ code, *C*, are defined as:

(1). The **left-symplectic dual** is defined as
  $$C^{{⊥}_{L_{s}}}=\left\{y\in E_{3}^{2n}|{\left(y,x\right)}_{s}=0,\forall \;x\in C\right\}.$$
(2). The **right-symplectic dual** of *C* is defined by
  $$C^{{⊥}_{R_{s}}}=\left\{y\in E_{3}^{2n}|{\left(x,y\right)}_{s}=0,\forall \;x\in C\right\}.$$

An $E_{3}$ code *C*, is called **left-symplectic self-dual** if $C^{{⊥}_{L_{s}}}=C$. Likewise, *C* is called **right-symplectic self-dual** if $C^{{⊥}_{R_{s}}}=C$. Then, we set $C^{{⊥}_{s}}=C^{{⊥}_{L_{s}}}\cap C^{{⊥}_{R_{s}}}$.

If any two codewords, x,y∈C, satisfy ${\left(x,y\right)}_{s}=0$, the code, *C*, is called a symplectic–self-orthogonal code. It is evident that *C* is symplectic–self-orthogonal if $C\subseteq C^{{⊥}_{s}}$.

A linear code, *C*, of length 2n is called left-symplectic-nice (resp. right-symplectic-nice) if it verifies $\left|C|\right|C^{{⊥}_{L_{s}}}|\;=9^{2n}$ (respectively, if $\left|C|\right|C^{{⊥}_{R_{s}}}|\;=9^{2n}$).

**Remark** **1.**  *In general, the relation “$\left|C|\right|C^{{⊥}_{R_{s}}}|\;=9^{2n}$” does not hold. For example, let C be an additive $E_{3}$ code of length 4 with (additive) generator matrix, G, where*

$$G=\left[\begin{array}{cccc} a & 2b & 0 & 0\\ 0 & 0 & a & 2b \end{array}\right]$$

*Let $\left(y_{1},y_{2},y_{3},y_{4}\right)$ be an arbitrary vector of $C^{{⊥}_{R_{s}}}$, then*

$${\left(\left(a,2b,0,0\right),\left(y_{1},y_{2},y_{3},y_{4}\right)\right)}_{s}=0\;and\;{\left(\left(0,0,a,2b\right),\left(y_{1},y_{2},y_{3},y_{4}\right)\right)}_{s}=0.$$

*These imply that, $ay_{3}+2by_{4}=0$ and $2ay_{1}+by_{2}=0$. Since ax=x and bx=x for any $x\in E_{3}$, $y_{3}+2y_{4}=0$ and $2y_{2}+y_{1}=0$. So, $y_{4}=y_{3}$ and $y_{2}=y_{1}$. Hence, $C^{{⊥}_{R_{s}}}=\left\{\left(y_{1},y_{1},y_{3},y_{3}\right)|\;y_{1},y_{3}\in E_{3}\right\}.$*

*It is easy to derive, |C|=9 and $|C^{{⊥}_{R_{s}}}|\;=9^{2}$. Thus, $\left|C|\right|C^{{⊥}_{R_{s}}}|\;=9^{3}<9^{4}$.*

## 3. Symplectic QSD and LCD $E_{3}$ Codes

In this section, first we define the notion symplectic QSD codes over the ring $E_{3}$ and then prove their multilevel construction from ternary linear codes. Further, we investigate the symplectic LCD codes and characterize free symplectic LCD code over $E_{3}$ in term of ternary generator matrix.

A symplectic–self-orthogonal $E_{3}$ code with length 2n and $9^{n}$ elements is called symplectic quasi self-dual (**symplectic QSD**).

In the following result, we establish a necessary and sufficient condition for a linear $E_{3}$ code to be self-orthogonal under the symplectic inner product.

**Lemma** **1.**  *A linear $E_{3}$ code C is symplectic–self-orthogonal if $\mathrm{res}\left(C\right)\subseteq \mathrm{tor}{\left(C\right)}^{{⊥}_{s}}$.*

**Proof.**  Suppose *C* is symplectic–self-orthogonal. Let x be an arbitrary codeword of res(C), then there exists xa+yc∈C, such that $\alpha_{E_{3}}\left(xa+yc\right)=x$. By definition, for any element z∈tor(C), zc∈C. From the symplectic–self-orthogonality of *C*, we have ${\left(xa+yc,zc\right)}_{s}={\left(x,z\right)}_{s}c=0$. This implies ${\left(x,z\right)}_{s}=0$. This means $z\in \mathrm{res}{\left(C\right)}^{{⊥}_{s}}$. Hence, $\mathrm{tor}\left(C\right)\subseteq \mathrm{res}{\left(C\right)}^{{⊥}_{s}}$. So, $\mathrm{res}\left(C\right)\subseteq \mathrm{tor}{\left(C\right)}^{{⊥}_{s}}$.

For the inverse assertion. Let $c,c^{\prime }\in C$. By Theorem 3.5 in [8], there exist $x,x^{\prime }\in \mathrm{res}\left(C\right)$ and $y,y^{\prime }\in \mathrm{tor}\left(C\right)$ such that c=xa+yc and $c^{\prime }=x^{\prime }a+y^{\prime }c$. For an arbitrary linear code, *C*, over $E_{3}$. Using a procedure similar to that given in [10], obtain $\mathrm{tor}{\left(C\right)}^{{⊥}_{s}}\subseteq \mathrm{res}{\left(C\right)}^{{⊥}_{s}}$ as res(C)⊆tor(C). Moreover, $\mathrm{res}\left(C\right)\subseteq \mathrm{tor}{\left(C\right)}^{{⊥}_{s}}\subseteq \mathrm{res}{\left(C\right)}^{{⊥}_{s}}$. This yields

$${\left(c,c^{\prime }\right)}_{s}={\left(xa+yc,x^{\prime }a+y^{\prime }c\right)}_{s}={\left(x,x^{\prime }\right)}_{s}a+{\left(x,y^{\prime }\right)}_{s}c=0.$$

Hence, *C* is symplectic–self-orthogonal over $E_{3}$. □

Next, we state the multilevel construction of symplectic QSD $E_{3}$ codes over from ternary linear codes.

**Theorem** **1.**  *Let B be a ternary linear code of length 2n. If B is a symplectic–self-orthogonal code, then the $E_{3}$ code, C, constructed through the relationship $C=Ba+B^{{⊥}_{s}}c$ is symplectic QSD. Furthermore, res(C)=B and $\mathrm{tor}\left(C\right)=B^{{⊥}_{s}}$.*

**Proof.**  We begin by proving that *C* is a linear $E_{3}$ code. Using the linearity of B and $B^{{⊥}_{s}}$, we note that *C* is closed under the addition operation. Let c∈C and $e\in E_{3}$, then $c=ba+b^{\prime }c$, where b∈B, $b^{\prime }\in B^{{⊥}_{s}}$ and by *c*-adic decomposition of *e*, we have $e=ra+r^{\prime }c$ where $r,r^{\prime }\in F_{3}$. So, we get

$$\begin{array}{cc} ce & =\left(ba+b^{\prime }c\right)\left(ra+r^{\prime }c\right)=bara+bar^{\prime }c+b^{\prime }cra+b^{\prime }cr^{\prime }c\\ & =bra^{2}+br^{\prime }ac+b^{\prime }rca+b^{\prime }r^{\prime }c^{2}\\ & =bra+br^{\prime }c. \end{array}$$

Therefore, $br,br^{\prime }\in B$ since the code B is linear, d∈B and $r,r^{\prime }\in F_{3}$. From the symplectic–orthogonality of B, we see that $br^{\prime }\in B^{{⊥}_{s}}$. We conclude that, ce∈C. Hence, *C* is a linear $E_{3}$ code.

Let $c=ba+b^{\prime }c,c^{\prime }=da+d^{\prime }c\in C$ for some b,d∈B and $b^{\prime },d^{\prime }\in B^{{⊥}_{s}}$. Then,

$$\begin{array}{cc} {\left(c,c^{\prime }\right)}_{s} & ={\left(ba+b^{\prime }c,da+d^{\prime }c\right)}_{s}\\ & ={\left(b,d\right)}_{s}a^{2}+{\left(b,d^{\prime }\right)}_{s}ac+{\left(b^{\prime },d\right)}_{s}ca+{\left(b^{\prime },d^{\prime }\right)}_{s}c^{2}\\ & ={\left(b,d\right)}_{s}a+{\left(b,d^{\prime }\right)}_{s}c=0. \end{array}$$

The last equality is derived because B is a symplectic–self-orthogonal code. Therefore, we have $C=Ba+B^{{⊥}_{s}}c$. This implies that $\left|C\right|=\left|B|\right|B^{{⊥}_{s}}|\;=9^{n}$. Consequently, *C* is a symplectic QSD code. Thus, the torsion and residue codes can be obtained by applying their definitions. □

The following result demonstrates that Theorem 1 does not hold if B is not a symplectic–self-orthogonal code.

**Theorem** **2.**  *For every $E_{3}$ code, C, given by $C=Ba+B^{{⊥}_{s}}c$ where B is a ternary linear code. Then, C is always an additive code over $E_{3}$, but never a linear code over $E_{3}$, unless B is symplectic–self-orthogonal.*

**Proof.**  First, we have to prove that *C* is an additive code over $E_{3}$. Let $c_{1},c_{2}\in C$, then $c_{1}=b_{1}a+b_{1}^{\prime }c$ and $c_{2}=b_{2}a+b_{2}^{\prime }c$, where $b_{1},b_{2}\in B$ and $b_{1}^{\prime },b_{2}^{\prime }\in B^{{⊥}_{s}}$. Thus, $c+c^{\prime }=\left(b_{1}+b_{2}\right)a+\left(b_{1}^{\prime }+b_{2}^{\prime }\right)c$. From the linearity of B and $B^{{⊥}_{s}}$ and the definition of *C*, it is easy to see that $c+c^{\prime }\in C$. Hence, *C* is an additive code over $E_{3}$.

Now, suppose that *C* is a linear code over $E_{3}$ and there exists a codeword b∈B. Then, a vector $b^{\prime }$ exists in $B^{{⊥}_{s}}$, such that $c=ba+b^{\prime }c\in C$. Thus, let $e\in E_{3}$; then, *e* has a *c*-adic decomposition form $e=ra+r^{\prime }c$ with $r,r^{\prime }\in F_{3}$. Further,

$$ce=\left(ba+b^{\prime }c\right)\left(ra+r^{\prime }c\right)=bra^{2}+br^{\prime }ac+b^{\prime }rca+b^{\prime }r^{\prime }c^{2}=\left(br\right)a+\left(br^{\prime }\right)c.$$

The last equality derived from $ca=c^{2}=0,a^{2}=a,ac=c$. Using the linearity of *C*, we have ce∈C and $br^{\prime }\in B^{{⊥}_{s}}$. Since $B^{{⊥}_{s}}$ is a ternary linear code, we have $br^{\prime }r^{\prime -1}=b\in B^{{⊥}_{s}}$, where $r^{\prime -1}$ denotes the multiplicative inverse of $r^{\prime }\in F_{3}$. This yields $B\subseteq B^{{⊥}_{s}}$. □

Next, we characterize the symplectic QSD codes over $E_{3}$ in terms of their associated ternary codes.

**Corollary** **1.**  *A linear $E_{3}$ code, C, of length 2n is symplectic QSD if $\mathrm{res}\left(C\right)=\mathrm{tor}{\left(C\right)}^{{⊥}_{s}}$.*

**Proof.**  For the direct statement, suppose *C* is symplectic QSD; then, $\left|C\right|=3^{2k_{1}+k_{2}}=3^{2n}$. This means $k_{1}=2n-\left(k_{1}+k_{2}\right)$. So, res(C) and $\mathrm{tor}{\left(C\right)}^{{⊥}_{s}}$ have the same dimension. Since *C* is symplectic–self-orthogonal, then by Lemma 1, $\mathrm{res}\left(C\right)\subseteq \mathrm{tor}{\left(C\right)}^{{⊥}_{s}}$. Hence, $\mathrm{res}\left(C\right)=\mathrm{tor}{\left(C\right)}^{{⊥}_{s}}$.

Conversely, since res(C)⊆tor(C), then from the given hypotheses, $\mathrm{res}\left(C\right)=\mathrm{tor}{\left(C\right)}^{{⊥}_{s}}$ and $\mathrm{tor}{\left(C\right)}^{{⊥}_{s}}\subseteq \mathrm{res}{\left(C\right)}^{{⊥}_{s}}$. These imply that, res(C) is ternary symplectic–self-orthogonal. By Theorem 2 and Theorem 3.5 in [8], the code, *C*, is symplectic QSD. □

The study of LCD codes with Euclidean inner product over non-unitary ring of order four first appeared in [4]. Further, the authors in [8] established right LCD $E_{3}$ codes. In addition, LCD codes under the symplectic inner product were introduced in [10]. In the following results, we extend this notion to codes over $E_{3}$.

A right-symplectic-nice code is said to be right-symplectic linear with complementary dual (**right-symplectic LCD**) if satisfies: $C\cap C^{{⊥}_{R_{s}}}=\left\{0\right\}$; *C* is right-symplectic LCD if $C⊕C^{{⊥}_{R_{s}}}=E_{3}^{2n}$.

Likewise, a left-symplectic-nice code is called left-symplectic linear with complementary dual (**left-symplectic LCD**) if satisfies $C\cap C^{{⊥}_{L_{s}}}=\left\{0\right\}$. Moreover, *C* is a left-symplectic LCD if $C⊕C^{{⊥}_{L_{s}}}=E_{3}^{2n}$.

**Remark** **2.**  *Note that non-trivial left-symplectic LCD code does not exist over $E_{3}$. If C≠{0} is a nonzero linear $E_{3}$ code of length 2n. Then, for any y∈C, yc∈C. We can see that yc=vc, where $v\in F_{3}^{2n}$. Since c is a left zero divisor, we have ${\left(yc,z\right)}_{s}=0$ for all z∈C. This shows $yc\in C^{{⊥}_{L_{s}}}$. Thus, $C\cap C^{{⊥}_{L_{s}}}\neq \left\{0\right\}$. Hence, the desired result achieved.*

Henceforward, we consider the right-symplectic LCD codes as symplectic LCD and therefore only the right (symplectic) dual.

Using part (i) of Theorem 4.10 in [8], Proposition 2.1 in [14] and by following Lemma 5 in [10], we state the next Lemma.

**Lemma** **2.**  *For any free linear $E_{3}$ code, C, of generator matrix, G, we have the following:*

*(1).*  *$C={\langle G_{3}a\rangle }_{E_{3}}$ where $G_{3}$ is a ternary matrix.*
*(2).*  *$C^{{⊥}_{R_{s}}}={\langle H_{3}\Omega a\rangle }_{E_{3}}$ where $H_{3}$ is a ternary parity check matrix related to $G_{3}$.*

**Proof.**  The proof of (1) is achieved in the proof of Theorem 4.10 in [8].

To prove (2), we have $C={\langle G_{3}a\rangle }_{E_{3}}$ for some ternary matrix $G_{3}$. Then, let $H_{3}$ be a parity check matrix of the linear code of generator matrix $G_{3}$. By Proposition 2.1 in [14], $H_{3}\Omega$ is a generator matrix of the symplectic dual of the ternary code whose generator matrix is $G_{3}$. Note that $\left(G_{3}a\right)\Omega {\left(H_{3}\Omega a\right)}^{T}=aG_{3}\Omega H_{3}^{T}a=0$. Hence, ${\langle H_{3}\Omega a\rangle }_{E_{3}}\subseteq C^{{⊥}_{R_{s}}}$.

For the converse inclusion, assume $z=\left(z_{1},z_{2},\cdots ,z_{2n}\right)\in C^{{⊥}_{R_{s}}}$ and $G_{3}$ is in standard form, i.e., $G_{3}=\left[I_{k}|M\right]$. Therefore, $G_{3}a=\left[I_{k}|M\right]a=\left[\begin{array}{c} r_{1}a\\ r_{2}a\\ \vdots \\ r_{k}a \end{array}\right]$ where $r_{1},\cdots ,r_{k}$ are the rows of $G_{3}$.

Furthermore, for all 0≤i≤k, we have ${\left(r_{i}a,z\right)}_{s}=0$. So, for any 1≤i≤k,

$$x_{i_{1}}az_{i_{1}}+x_{i_{2}}az_{i_{2}}+\cdots +x_{i_{t}}az_{i_{t}}=0,$$

for some 1≤t≤k and $x_{i_{j}}$ are nonzero elements of $F_{3}$. Since ax=x for all $x\in E_{3}$, we get

$$x_{i_{1}}z_{i_{1}}+x_{i_{2}}z_{i_{2}}+\cdots +x_{i_{t}}z_{i_{t}}=0.$$

Further, we obtain the next *k* equations:

$$\begin{array}{c} \beta_{1}z_{n+1}+0+0+\cdots +0+x_{k+i_{1}}z_{k+i_{1}}+\cdots +x_{i_{l_{1}}}z_{i_{l_{1}}}=0,\\ 0+\beta_{2}z_{n+2}+0+\cdots +0+x_{k+i_{2}}z_{k+i_{2}}+\cdots +x_{i_{l_{2}}}z_{i_{l_{2}}}=0,\\ \vdots \\ 0+0+\cdots +0+\beta_{k}z_{k}+x_{k+i_{k}}z_{k+i_{k}}+\cdots +x_{k+i_{k}}z_{i_{k}}=0 \end{array}$$

where for each 0≤t≤k,  ${\beta_{t}}^{’}s$ are 1 or 2 and the indices $k+i_{t},\cdots \;i_{l_{t}}$ of **z** correspond to the nonzero positions of $r_{t}a$. Moreover, there are at most 2n−k free variables in the previous *k* equations, then there are $9^{2n-k}$ solutions of **z**. This implies that $|C^{{⊥}_{R_{s}}}|\leq 9^{2n-k}.$ Since ${\langle H_{3}\Omega a\rangle }_{E_{3}}=9^{2n-k}$ and ${\langle H_{3}\Omega a\rangle }_{E_{3}}\subseteq C^{{⊥}_{R_{s}}}$, we conclude that $C^{{⊥}_{R_{s}}}={\langle H_{3}\Omega a\rangle }_{E_{3}}$. □

**Proposition** **1.**  *Let C be a linear $E_{3}$ code having length 2n. Then, C is a free code if it is right-symplectic-nice.*

**Proof.**  We have *C* is a linear code over $E_{3}$, then $\left|C|\right|C^{{⊥}_{R_{s}}}|\;=3^{2k_{1}+k_{2}}\times 9^{2n-k_{1}}=3^{4n+k_{2}}$. Hence, *C* is right-symplectic-nice if $k_{2}=0$. □

**Corollary** **2.**  *Every symplectic LCD code, C, over $E_{3}$ is free.*

**Proof.**  Using the definition of symplectic LCD code, we found that *C* is right-symplectic-nice. By Proposition 1 the code, *C*, is free. □

Now, we construct symplectic LCD codes over $E_{3}$ from ternary symplectic LCD codes.

**Proposition** **2.**  *Let B be a symplectic LCD ternary [2n,k] code having a generator matrix $G_{3}$. Then, the $E_{3}$ code $C={\langle G_{3}a\rangle }_{E_{3}}$ is a symplectic LCD $E_{3}$ code.*

**Proof.**  We have $C={\langle G_{3}a\rangle }_{E_{3}}$; then, by statement (2) of Lemma 2, $C^{{⊥}_{R_{s}}}={\langle H_{3}\Omega a\rangle }_{E_{3}}$ where $H_{3}$ is a parity check matrix of the matrix $G_{3}$ and $H_{3}\Omega$ is a generator matrix of the ternary symplectic dual code related to $G_{3}$.

Further, $\left|C|\right|C^{{⊥}_{R_{s}}}|\;=3^{2k}\times 9^{2n-k}=9^{2n}$. This implies that *C* is right-symplectic-nice.

To prove that $C\cap C^{{⊥}_{R_{s}}}=\left\{0\right\}$, assume (if possible) 0≠z in $C\cap C^{{⊥}_{R_{s}}}$. Then, there are two possibilities:

(1). Let z=yc where $0\neq y\in F_{3}^{2n}$ and $G_{3}$ is in standard form. By investigation of the matrix $G_{3}a$, we conclude that y can be expressed as a sum of scalar multiples of some rows of $G_{3}$. Hence, y∈B. Likewise, we can prove that $y\in B^{{⊥}_{s}}$.
  Consequently, $0\neq y\in B\cap B^{{⊥}_{s}}$, this contradicts with B is a ternary symplectic LCD code.
(2). Suppose z is not a product of a ternary vector by *c*, then
  $$z=\sum g_{i}au_{i}\;\mathrm{and}\;z=\sum g_{j}av_{j}.$$
  where $g_{j}a$ is a row of $G_{3}a$ for some (distinct) *i* and $u_{i}\in E_{3}$ and $g_{j}a$ is a row of $H_{3}\Omega a$ for some (distinct) *j* and $v_{i}\in E_{3}$. Therefore,
  $$z=\sum g_{i}u_{i}\;\mathrm{also}\;z=\sum g_{j}v_{j}$$
  where $g_{i}$ is a row of $G_{3}$ for some (distinct) *i*, *j* and $u_{i},v_{i}\in E_{3}$.
  From the multiplication table of $E_{3}$, we have $za=\sum g_{i_{t}}$ and $z=\sum g_{j_{l}}$, where $g_{i_{t}}$ is a row of $G_{3}$ for some distinct $i_{t}$ and $g_{j_{l}}$ is a row of $H_{3}\Omega$ for some distinct $j_{l}$.
  Therefore, $\sum g_{i_{t}}a-\sum g_{j_{l}}a=\left(\sum g_{i_{t}}-\sum g_{j_{l}}\right)a=0$. This implies that, $\sum g_{i_{t}}-\sum g_{j_{l}}=0$, so $\sum g_{i_{t}}=\sum g_{j_{l}}\in B\cap B^{{⊥}_{s}}$. This implies that $B\cap B^{{⊥}_{s}}\neq \left\{0\right\},$ which contradicts the fact that B is a symplectic LCD code.

□

The example below presents a symplectic LCD $E_{3}$ code constructed according to Proposition 2.

**Example** **1.**  *Let B be the ternary [4, 2] code of generator matrix $G_{3}=\left[\begin{array}{cccc} 1 & 0 & 1 & 1\\ 0 & 1 & 2 & 1 \end{array}\right]$.*

*Since $G_{3}\Omega G_{3}^{T}=\left[\begin{array}{cc} 0 & 1\\ 2 & 0 \end{array}\right]$ is a non-singular matrix over $F_{3}$; then, by Theorem 1 in [15], B is a ternary symplectic LCD code of length *4*. Then, by Proposition 2, ${\langle G_{3}a\rangle }_{E_{3}}$ is a symplectic LCD $E_{3}$ code.*

*Now, we verify the conditions of the definition of symplectic LCD $E_{3}$ code to prove that the code ${\langle G_{3}a\rangle }_{E_{3}}$ is symplectic LCD over $E_{3}$.*

*Assume, $C={\langle G_{3}a\rangle }_{E_{3}}={\langle \left[\begin{array}{cccc} a & 0 & a & a\\ 0 & a & 2a & a \end{array}\right]\rangle }_{E_{3}}$. Then, C can be represented by the set*

$$C=\left\{\left(ax,ax^{\prime },ax+2ax^{\prime },ax+ax^{\prime }\right)|\;x,x^{\prime }\in E_{3}\right\}=\left\{\left(x,x^{\prime },x+2x^{\prime },x+x^{\prime }\right)|\;x,x^{\prime }\in E_{3}\right\}.$$

*For any $(y,y^{\prime },z,z^{\prime })\in C$, we have*

$${\left(\left(a,0,a,a\right),\left(y,y^{\prime },z,z^{\prime }\right)\right)}_{s}=0\;and\;{\left(\left(0,a,2a,a\right),\left(y,y^{\prime },z,z^{\prime }\right)\right)}_{s}=0.$$

*These imply, $az+2ay+2ay^{\prime }=0$ and $az^{\prime }+ay+2ay^{\prime }=0$. Thus, $z+2y+2y^{\prime }$ and $z^{\prime }+y+2y^{\prime }=0$. So, $z=y+y^{\prime }$ and $z^{\prime }=2y+y^{\prime }$. Hence,*

$$C^{{⊥}_{R_{s}}}=\left\{\left(y,y^{\prime },y+y^{\prime },2y+y^{\prime }\right)|\;y,y^{\prime }\in E_{3}\right\}.$$

*Clearly, $|C^{{⊥}_{R_{s}}}|\;=9^{2}$, $\left|C\right|=9^{2}$. Then, $\left|C|\right|C^{{⊥}_{R_{s}}}|\;=9^{4}$. Furthermore, $C\cap C^{{⊥}_{R_{s}}}=\left\{0\right\}$. Therefore, C is symplectic LCD $E_{3}$ code.*

The inverse statement of Proposition 2 is partially true, as the following proposition states.

**Proposition** **3.**  *A free symplectic LCD $E_{3}$ code, C, is the $E_{3}$-span by the matrix Ga with G is a generator matrix of a ternary symplectic LCD code B.*

**Proof.**  Since *C* is free, then by part (1) of Lemma 2, *C* is the $E_{3}$-span by the matrix Ga where *G* is a generator matrix of the ternary code B. Moreover, from part (2) of Lemma 2, $C^{{⊥}_{R_{s}}}$ is generated by the matrix HΩa, where *H* is a parity check matrix of B. Since *C* is symplectic LCD, $C\cap C^{{⊥}_{R_{s}}}=\left\{0\right\}$. Further, suppose $\left(0\neq x\right)\in B\cap B^{{⊥}_{R_{s}}}$, then $xa\in C\cap C^{{⊥}_{R_{s}}}$; this contradicts $C\cap C^{{⊥}_{R_{s}}}=\left\{0\right\}$. Therefore, B is a ternary symplectic LCD code. □

**Corollary** **3.**  *For any symplectic LCD code, C, over $E_{3}$, the ternary codes tor(C) and res(C) are symplectic LCD codes.*

**Proof.**  Since *C* is symplectic LCD code, then by Corollary 2, *C* is free. Then, by Proposition 3, *C* is the $E_{3}$-span by the matrix Ga where *G* is a generator matrix of ternary symplectic LCD code. From the freeness of *C*, we have res(C)=tor(C) and *G* will be a generator matrix for both res(C) and tor(C). □

## 4. Symplectic ACD Codes

In this section, we introduce the right-symplectic-nice and left-symplectic-nice additive codes, as well as right-symplectic ACD and left-symplectic ACD $E_{3}$ codes. We provide some criteria for the existence of the right-symplectic ACD $E_{3}$ codes. Moreover, for additive, linear and free $E_{3}$ codes, in terms of their torsion and residue codes, we also determine the residue and torsion codes of the right-symplectic dual of such codes. At the end, we show that any linear $E_{3}$ code is free if, and only if, its right and left-symplectic duals have the same residue code.

An additive $E_{3}$ code of length 2n is called right-symplectic-nice (respectively, left-symplectic-nice) code if $\left|C|\right|C^{{⊥}_{R_{s}}}|\;=9^{2n}$ (respectively, $\left|C|\right|C^{{⊥}_{L_{s}}}|\;=9^{2n}$).

A right-symplectic-nice additive $E_{3}$ code, *C*, is called right-symplectic additive complementary dual (**right-symplectic ACD**) if $C\cap C^{{⊥}_{R_{s}}}=\left\{0\right\}$.

Likewise, a left-symplectic-nice additive $E_{3}$ code, *C*, is called left-symplectic additive complementary dual (**left-symplectic ACD**) if $C\cap C^{{⊥}_{L_{s}}}=\left\{0\right\}$.

**Lemma** **3.**  *Let C be an additive $E_{3}$ code. Then, the right-symplectic dual of C is a free linear $E_{3}$ code and its left-symplectic dual is a non-free linear $E_{3}$ code.*

**Proof.**  Firstly, we have to prove that $C^{{⊥}_{R_{s}}}$ and $C^{{⊥}_{L_{s}}}$ are linear codes over $E_{3}$. Let $z,z^{\prime }\in C^{{⊥}_{R_{s}}}$ and y∈C. Then,

$${\left(y,z+z^{\prime }\right)}_{s}={\left(y,z\right)}_{s}+{\left(y,z^{\prime }\right)}_{s}=0+0=0.$$

Hence, $z+z^{\prime }\in C^{{⊥}_{R_{s}}}$. Thus, for any $z\in C^{{⊥}_{R_{s}}}$, $u\in E_{3}$ and y∈C, we have

$${\left(y,zr\right)}_{s}={\left(y,z\right)}_{s}r=0.$$

So, $zr\in C^{{⊥}_{R_{s}}}$. Further, $C^{{⊥}_{R_{s}}}$ is a right submodule of $E_{3}^{2n}$. This shows that $C^{{⊥}_{R_{s}}}$ is a linear $E_{3}$ code.

Next, we prove that $C^{{⊥}_{R_{s}}}$ is free code. Let $z=uc\in C^{{⊥}_{R_{s}}}$ where $u\in F_{3}^{2n}$. Then according to multiplication table of $E_{3}$, for any y∈C, we get ${\left(y,z\right)}_{s}={\left(y,uc\right)}_{s}=tc=0$. This implies, t is multiple of 3. Therefore, ${\left(y,ua\right)}_{s}={\left(y,ua\right)}_{s}=ta=0$. This means $ua\in C^{{⊥}_{R_{s}}}$. By Theorem 4.12 in [8], consequently $C^{{⊥}_{R_{s}}}$ is free.

Now, we have to prove $C^{{⊥}_{L_{s}}}$ is also linear over $E_{3}$. Let $x,x^{\prime }\in C^{{⊥}_{L_{s}}}$ and y∈C. Then,

$${\left(x+x^{\prime },y\right)}_{s}={\left(z,y\right)}_{s}+{\left(z^{\prime },y\right)}_{s}=0+0=0.$$

So, $x+x^{\prime }\in C^{{⊥}_{L_{s}}}$. Therefore, let $x\in C^{{⊥}_{R_{s}}}$, y∈C and let $r\in E_{3}$, then $r=ea+e^{\prime }c$ where $e,e^{\prime }\in F_{3}$. Thus,

$${\left(xr,y\right)}_{s}={\left(x\left(ea+e^{\prime }c\right),y\right)}_{s}={\left(xea,y\right)}_{s}+{\left(xe^{\prime }c,y\right)}_{s}.$$

Since ${\left(xa,y\right)}_{s}={\left(xb,y\right)}_{s}={\left(x,y\right)}_{s}=0$, we get ${\left(xea,y\right)}_{s}+{\left(xe^{\prime }c,y\right)}_{s}={\left(xe,y\right)}_{s}+0={\left(x,y\right)}_{s}e=0$. So, ${\left(xr,y\right)}_{s}=0$. Hence, $xr\in C^{{⊥}_{L_{s}}}$. Consequently, $C^{{⊥}_{L_{s}}}$ is a linear $E_{3}^{2n}$ code.

It remains to prove $C^{{⊥}_{L_{s}}}$ is not a free code. Suppose $C^{{⊥}_{L_{s}}}$ is free over $E_{3}$. Since *c* is left zero divisor, we get ${\langle cI_{2n}\rangle }_{F_{3}}\subseteq C^{{⊥}_{L_{s}}}$. Thus, according to Theorem 4.12 in [8], ${\langle aI_{2n}\rangle }_{F_{3}}\subseteq C^{{⊥}_{L_{s}}}$. These yield $|C^{{⊥}_{L_{s}}}|\geq 9^{n}\times 9^{n}=9^{2n}$. Since $C^{{⊥}_{L_{s}}}\subseteq E_{3}^{2n}$, we have $|C^{{⊥}_{L_{s}}}|\leq 9^{2n}$. Hence, $C^{{⊥}_{L_{s}}}=E_{3}^{2n}$. This implies that C={0}, which is impossible. Hence, $C^{{⊥}_{L_{s}}}$ is not a free $E_{3}$ code. □

**Theorem** **3.**  *Let C be a linear code over $E_{3}$ of length 2n. Then, $C={\left(C^{{⊥}_{R_{s}}}\right)}^{{⊥}_{R_{s}}}$ if C is right-symplectic-nice.*

**Proof.**  Assume, ${\left(C^{{⊥}_{R_{s}}}\right)}^{{⊥}_{R_{s}}}=C$. By Lemma 3, $C^{{⊥}_{R_{s}}}$ is a free linear $E_{3}$ code. Thus, from Proposition 1, $C^{{⊥}_{R_{s}}}$ is right-symplectic-nice. This yields $|C^{{⊥}_{R_{s}}}\left|\right|{\left(C^{{⊥}_{R_{s}}}\right)}^{{⊥}_{R_{s}}}\left|\;=\;|C|\right|C^{{⊥}_{R_{s}}}|\;=9^{2n}$. Hence, *C* is right-symplectic-nice.

Conversely, assume *C* is right-symplectic-nice. From Proposition 1, we have *C* is free. Furthermore, by Lemma 3, $C^{{⊥}_{R_{s}}}$ is free. Thus, by Lemma 2, for any y∈C and $z\in C^{{⊥}_{R_{s}}}$, we assume y=la and z=ta for some $l,t^{\prime }\in F_{3}^{2n}$. Then, $0={\left(y,z\right)}_{s}={\left(la,ta\right)}_{s}={\left(l,t\right)}_{s}a^{2}={\left(l,t\right)}_{s}a=2{\left(t,l\right)}_{s}a=2{\left(z,y\right)}_{s}$. So, ${\left(z,y\right)}_{s}=0$, for any $z\in C^{{⊥}_{R_{s}}}$. Then, $y\in {\left(C^{{⊥}_{R_{s}}}\right)}^{{⊥}_{R_{s}}}$. This shows that $C\subseteq {\left(C^{{⊥}_{R_{s}}}\right)}^{{⊥}_{R_{s}}}$. Furthermore, *C*, $C^{{⊥}_{R_{s}}}$ are both right-symplectic-nice, which means $\left|C|\right|C^{{⊥}_{R_{s}}}\left|\;=\;\right|{\left(C^{{⊥}_{R_{s}}}\right)}^{{⊥}_{R_{s}}}\left|\right|C^{{⊥}_{R_{s}}}|$. So, ${\left(C^{{⊥}_{R_{s}}}\right)}^{{⊥}_{R_{s}}}\left|\;=\;|C|\right|$. This together with $C\subseteq {\left(C^{{⊥}_{R_{s}}}\right)}^{{⊥}_{R_{s}}}$ imply that $C={\left(C^{{⊥}_{R_{s}}}\right)}^{{⊥}_{R_{s}}}$. □

The next result characterizes the one-sided duals of a symplectic LCD $E_{3}$ code.

**Proposition** **4.**  *Let C be a symplectic LCD $E_{3}$ code, then $C^{{⊥}_{R_{s}}}$ is symplectic LCD but $C^{{⊥}_{L_{s}}}$ is not.*

**Proof.**  Suppose *C* is symplectic LCD code. Then, *C* is right-symplectic-nice with $C\cap C^{{⊥}_{R_{s}}}=\left\{0\right\}$. By Theorem 3, ${\left(C^{{⊥}_{R_{s}}}\right)}^{{⊥}_{R_{s}}}=C$. Thus, $|C^{{⊥}_{R_{s}}}\left|\right|{\left(C^{{⊥}_{R_{s}}}\right)}^{{⊥}_{R_{s}}}\left|\;=\;\right|C^{{⊥}_{R_{s}}}\left||C\right|=9^{2n}$. Furthermore, $C^{{⊥}_{R_{s}}}\cap {\left(C^{{⊥}_{R_{s}}}\right)}^{{⊥}_{R_{s}}}=C^{{⊥}_{R_{s}}}\cap C=0$. These imply that $C^{{⊥}_{R_{s}}}$ is a symplectic LCD $E_{3}$ code.

Further, from Lemma 3, $C^{{⊥}_{L_{s}}}$ is non-free linear $E_{3}$ code. Hence, by Proposition 1, $C^{{⊥}_{L_{s}}}$ is not right-symplectic-nice. So, $C^{{⊥}_{L_{s}}}$ is not symplectic LCD. □

**Proposition** **5.**  *If C is a right-symplectic ACD $E_{3}$ code with $3^{t}$ elements then t is even.*

**Proof.**  By Lemma 3, $C^{{⊥}_{R_{s}}}$ is free. This yields, $C^{{⊥}_{R_{s}}}=9^{k_{1}}=3^{2k_{1}}$. Since *C* is right-symplectic ACD, then $\left|C|\right|C^{{⊥}_{R_{s}}}|\;=9^{2n}$. Thus, $\left|C\right|=3^{4n-2k_{1}}$. Hence, $t=4n-2k_{1}$; this means *t* is even. □

Next, we construct the right-symplectic ACD codes over $E_{3}$.

**Proposition** **6.**  *Let C be an additive $E_{3}$ code of length 2n with $C\cap C^{{⊥}_{R_{s}}}=Cc\cap C^{{⊥}_{R_{s}}}=\left\{0\right\}$ and $|C^{{⊥}_{R_{s}}}||C|<9^{2n}$. Then, for any nonzero element $y=z+z^{\prime }c\in C+Cc$, if $y\in C^{{⊥}_{R_{s}}}$, then z is a multiple of a ternary vector by c, z≠0, $z^{\prime }c\neq 0$ and z∈C∖Cc.*

**Proof.**  Let $0\neq y=z+z^{\prime }c\in C+Cc$, where $z,z^{\prime }\in C$. We suppose that $y\in C^{{⊥}_{R_{s}}}$. Then, we get the following cases:

(1). If z=0 and $z^{\prime }c=0$, then y=0 which is not possible.
(2). If z=0 and $z^{\prime }c\neq 0$, then $y=z^{\prime }c\in C^{{⊥}_{R_{s}}}$. This implies that, $0\neq y\in Cc\cap C^{{⊥}_{R_{s}}}=\left\{0\right\}$ which is not possible.
(3). If z≠0 and $z^{\prime }c=0$, then $y=z\in C^{{⊥}_{R_{s}}}$. This shows that $0\neq y\in C\cap C^{{⊥}_{R_{s}}}=0$ which leads to a contradiction.
(4). If z≠0 and $z^{\prime }c\neq 0$, then $y=z+z^{\prime }c$. Suppose, z is not a multiple of a ternary vector by *c*. So, zc≠0. By Lemma 3, $C^{{⊥}_{R_{s}}}$ is a free linear $E_{3}$ code. This together with $y\in C^{{⊥}_{R_{s}}}$, we obtain $yc=\left(z+z^{\prime }c\right)c=zc\in C^{{⊥}_{R_{s}}}$. Thus, $zc\in Cc\cap C^{{⊥}_{R_{s}}}=\left\{0\right\}$ which is not possible. Hence, z is a multiple of a ternary vector by *c*. Further, if z∈Cc, then y∈Cc, also $y\in Cc\cap C^{{⊥}_{R_{s}}}=\left\{0\right\}$. It again leads to a contradiction. Hence, z∈C∖Cc.

By (1), (2), (3), and (4), if $0\neq y=z+z^{\prime }c\in C^{{⊥}_{R_{s}}}$, then z≠0, $z^{\prime }c\neq 0$, z is a multiple of a ternary vector by *c*, and z∈C∖Cc. This completes the proof. □

**Theorem** **4.**  *Let C be an additive $E_{3}$ code of length 2n. If $\left|C|\right|C^{{⊥}_{R_{s}}}|<9^{2n}$ and $C\cap C^{{⊥}_{R_{s}}}=Cc\cap C^{{⊥}_{R_{s}}}=\left\{0\right\}$, then C can be extended to a right-symplectic ACD code over $E_{3}$ by adding some elements of Cc.*

**Proof.** (1). First, we show that Cc⊈C. Suppose $B={\langle C\rangle }_{E_{3}}=Ca+Cc=\left\{za+z^{\prime }c|\;z,z^{\prime }\in C\setminus Cc\right\}$.

Since *c* is a left zero-divisor, we suppose that z and $z^{\prime }$ are not multiples of a ternary vectors by *c*. Clearly, B is a free linear $E_{3}$ code and $B^{{⊥}_{R_{s}}}=C^{{⊥}_{R_{s}}}$. From Lemma 2 and Proposition 2, B is right-symplectic-nice.
  If Cc⊆C, according to [8], we define a map $\pi_{1}:C\to B$ by
  $$\pi_{1}\left(0\right)=0,\;\pi_{1}\left(z\right)=za\;\mathrm{and}\;\pi_{1}\left(zc\right)=zc,\;\mathrm{for}\;\mathrm{any}\;z\in C\setminus Cc.$$
  Note that, for any $z,z^{\prime }\in C$, $\pi_{1}\left(z+z^{\prime }\right)=\pi_{1}\left(z\right)+\pi_{1}\left(z^{\prime }\right)$. It is clear that $\pi_{1}$ is a surjective linear mapping over $F_{3}$. Thus, |C|≥|B| since $\pi_{1}$ is surjective. Thus, $\left|C|\right|C^{{⊥}_{R_{s}}}\left|\;\geq |B|\right|C^{{⊥}_{R_{s}}}\left|\;=|B|\right|B^{{⊥}_{R_{s}}}|\;=9^{2n}$. So, $\left|C|\right|C^{{⊥}_{R_{s}}}|\geq 9^{2n}$ which contradicts the hypothesis. Hence, Cc⊈C. Moreover, *C* can be extended to a right-symplectic-nice code by adding some codewords of Cc.

(2). Now, we prove that a vector $z^{\prime }c$ exists in Cc∖C such that $z+z^{\prime }c\notin C^{{⊥}_{R_{s}}}$ for any z∈C. Assume this is not true, i.e., there is a codeword z of *C* such that $z+z^{\prime }c\in C^{{⊥}_{R_{s}}}$ for any $z^{\prime }c\in Cc\setminus C$. Then, by Proposition 6, a ternary vector y exists such that z=yc and z in C∖Cc.
  Suppose for any $z_{1}^{\prime }c,z_{2}^{\prime }c\in C\setminus Cc$ with $z_{1}^{\prime }c\neq z_{2}^{\prime }c$, there are $z_{1},z_{2}\in C$ such that $z_{1}+z_{1}^{\prime }c,z_{2}+z_{2}^{\prime }c\in C^{{⊥}_{R_{s}}}$.
  If possible, let $z_{1}=z_{2}$, then $\left(z_{1}^{\prime }+2z_{2}^{\prime }\right)c=\left(z_{1}+z_{1}^{\prime }c\right)+2\left(z_{2}+z_{2}^{\prime }c\right)\in C^{{⊥}_{R_{s}}}$ (since $C^{{⊥}_{R_{s}}}$ is a linear code over $E_{3}$ and $z_{1}+z_{1}^{\prime }c,z_{2}+z_{2}^{\prime }c\in C^{{⊥}_{R_{s}}}$).
  Thus, $z_{1}^{\prime }+2z_{2}^{\prime }\in C$ as $z_{1}^{\prime },z_{2}^{\prime }\in C$. This yields $(z_{1}^{\prime }+2z_{2}^{\prime })c\in Cc$. So, $\left(z_{1}^{\prime }+2z_{2}^{\prime }\right)c\in Cc\cap C^{{⊥}_{R_{s}}}=\left\{0\right\}$. Therefore, $z_{1}^{\prime }c=z_{2}^{\prime }c$ which is not possible.
  Hence, we have $z_{1}\neq z_{2}$. Let us define $\pi_{2}:B\to C$. We will discuss the following cases:
  (2.1). For any z∈C∖Cc, if zc∈Cc∖C, we define $\pi_{2}$ as
    $$\pi_{2}\left(0\right)=0,\;\pi_{2}\left(\mathrm{za}\right)=z\;\mathrm{and}\;\pi_{2}\left(zc\right)=y.$$
    where, y is the given vector satisfies $y+za\in C^{{⊥}_{R_{s}}}$. For all $z,z^{\prime }\in B$, $\pi_{2}\left(z+z^{\prime }\right)=\pi_{2}\left(z\right)+\pi_{2}\left(z^{\prime }\right)$. Thus, it is easy to see that $\pi_{2}$ is injective linear map over $F_{3}$. Therefore, if $\pi_{2}\left(zc\right)\neq \pi_{2}\left(z^{\prime }c\right)$, then $y\neq y^{\prime }$, as for $zc,z^{\prime }c\in Cc$, there exist $y,y^{\prime }\in C$ such that $y+zc,y+zc\in C^{{⊥}_{R_{s}}}$ with $y\neq y^{\prime }$.
  (2.2). For any z∈C∖Cc, if zc∈Cc∖C, we define $\pi_{2}$ by
    $$\pi_{2}\left(0\right)=0,\;\pi_{2}\left(\mathrm{za}\right)=z\;\mathrm{and}\;\pi_{2}\left(zc\right)=zc.$$
    For all $z,z^{\prime }\in B$, $\pi_{2}\left(z+z^{\prime }\right)=\pi_{2}\left(z\right)+\pi_{2}\left(z^{\prime }\right)$. Furthermore, $\pi_{2}$ is injective $F_{3}$-linear map.
  For both cases, since $\pi_{2}$ is injective, we have |C|≥|B|. This yields $\left|C|\right|C^{{⊥}_{R_{s}}}\left|\geq |B|\right|B^{{⊥}_{R_{s}}}|=9^{2n}$. So, $\left|C|\right|C^{{⊥}_{R_{s}}}|\geq 9^{2n}$, this is a contradiction.
  Hence, $z^{\prime }c\in Cc\setminus C$ such that $z+z^{\prime }c\notin C^{{⊥}_{R_{s}}}$ for any z∈C.

At this time, we add zc to *C* to get a new code *D*. We can check that, $D\cap D^{{⊥}_{R_{s}}}=\left\{0\right\}$. If *D* is right-symplectic-nice, then *D* is right-symplectic ACD. Otherwise, repeat steps (1) and (2) for *D*. □

The definitions of the associated ternary codes (i.e., residue and torsion codes) of a linear $E_{3}$ code can be extended into an additive $E_{3}$ code *C*. Clearly, these codes are linear codes. Consider, $\dim\;\left(\mathrm{res}\left(C\right)\right)=d_{1}$ and $\dim\;\left(\mathrm{tor}\left(C\right)\right)=d_{2}$.

For a linear $E_{3}$ code, *C*, of generator matrix *G* in the form given in Corollary 4.11 in [8]. Then, $\dim\;(\mathrm{res}\left(C\right)=k_{1}=d_{1}$ and $\dim\;\left(\mathrm{tor}\left(C\right)\right)=k_{1}+k_{2}=d_{1}+k_{2}=d_{2}$, where $k_{2}$ is the $F_{3}$-dimension of the set of all codewords of *C* which are scalar multiples of $c\in E_{3}$ and which can not be obtained from the upper two blocks of *G*.

For any codeword r of the code, *C*, we can write it in *c*-adic decomposition form as r=ua+vc with u,v are ternary vectors, so that $\alpha_{E_{3}}\left(r\right)=u$.

**Theorem** **5.** *For any additive $E_{3}$ code, C, of length 2n. We have*

$$C^{{⊥}_{R_{s}}}={\langle \mathrm{res}{\left(C\right)}^{{⊥}_{s}}\rangle }_{E_{3}}\;and\;\left|C^{{⊥}_{R_{s}}}\right|=9^{2n-\dim\;(\mathrm{res}(C))}=9^{2n-d_{1}}.$$

**Proof.**  For any z∈C, there exists v∈res(C) such that za=va. Let $u\in \mathrm{res}{\left(C\right)}^{{⊥}_{R_{s}}}$. Then,

$${\left(z,ua\right)}_{s}={\left(za,ua\right)}_{s}={\left(va,ua\right)}_{s}={\left(v,u\right)}_{s}a^{2}={\left(v,u\right)}_{s}a=0.$$

$${\left(z,ub\right)}_{s}={\left(za,ub\right)}_{s}={\left(va,ub\right)}_{s}={\left(vb,ub\right)}_{s}={\left(v,u\right)}_{s}b=0.$$

These show that $ua,ub\in C^{{⊥}_{R_{s}}}$. Hence, ${\langle \mathrm{res}{\left(C\right)}^{{⊥}_{s}}\rangle }_{E_{3}}\subseteq C^{{⊥}_{R_{s}}}$ since *a* and *b* are generators of $E_{3}$ and $ua,ua\in C^{{⊥}_{R_{s}}}$. Clearly, ${\langle \mathrm{res}{\left(C\right)}^{{⊥}_{s}}\rangle }_{E_{3}}={\langle \mathrm{res}\left(C\right)\rangle }_{E_{3}}^{{⊥}_{Rs}}$.

Now, we have to prove $C^{{⊥}_{R_{s}}}\subseteq {\langle \mathrm{res}{\left(C\right)}^{{⊥}_{s}}\rangle }_{E_{3}}$. Let x∈res(C), then there exists $xa+yc\in C^{{⊥}_{R_{s}}}$ such that $\alpha_{E_{3}}\left(xa+yc\right)=x$.

Since *c* is a left-zero divisor, then for any $z\in C^{{⊥}_{R_{s}}}$, we get

$${\left(xa,z\right)}_{s}={\left(xa+yc,z\right)}_{s}=0\;\mathrm{and}\;{\left(xb,z\right)}_{s}={\left(xa,z\right)}_{s}=0.$$

This yields $z\in {\langle \mathrm{res}\left(C\right)\rangle }_{E_{3}}^{{⊥}_{R_{s}}}$. So, $C^{{⊥}_{R_{s}}}\subseteq {\langle \mathrm{res}\left(C\right)\rangle }_{E_{3}}^{{⊥}_{R_{s}}}$.

Consequently, $C^{{⊥}_{R_{s}}}={\langle \mathrm{res}{\left(C\right)}^{{⊥}_{s}}\rangle }_{E_{3}}$. Further, $|C^{{⊥}_{R_{s}}}\left|\;=\;\right|{\langle \mathrm{res}{\left(C\right)}^{{⊥}_{s}}\rangle }_{E_{3}}|\;=9^{2n-d_{1}}$. □

The following result derives a sufficient condition for an additive $E_{3}$ code to be right-symplectic ACD.

**Proposition** **7.**  *Let C be an additive $E_{3}$ code of length 2n. Then, C is a right-symplectic ACD code if the next statements hold:*

*(1)*. *The ternary code res(C) is symplectic LCD.*
*(2)*. *$\mathrm{tor}\left(C\right)c\cap C^{{⊥}_{R_{s}}}=\left\{0\right\}$.*
*(3)*. *$3^{d_{1}+d_{2}}\times 9^{2n-d_{1}}=9^{2n}$ (i.e., $d_{1}=d_{2}$).*

**Proof.**  First, it is clear that, $\left|C\right|=\left|\mathrm{res}\left(C\right)||\mathrm{tor}\left(C\right)\right|=3^{d_{1}+d_{2}}$ and by Theorem 5, $|C^{{⊥}_{R_{s}}}|\;=9^{2n-d_{1}}$. So, $\left|C|\right|C^{{⊥}_{R_{s}}}|\;=9^{2n}$. This demonstrates that *C* is a right-symplectic-nice $E_{3}$ code. The final step is to show that $C\cap C^{{⊥}_{R_{s}}}=\left\{0\right\}$. If it is possible, let $0\neq z=\left(z_{1},z_{2},\cdots ,z_{2n}\right)\in C\cap C^{{⊥}_{R_{s}}}$. As $\mathrm{tor}\left(C\right)c\cap C^{{⊥}_{R_{s}}}=\left\{0\right\}$, z is not a multiple of ternary vector by *c*. This yields $\alpha_{E_{3}}\left(z\right)\neq 0$. Thus, for any $y=(y_{1},y_{2},\cdots ,y_{2n})\in C$, we have ${\left(y,z\right)}_{s}=0$. As $\alpha_{E_{3}}$ is a (ring) morphism, we get

$$\begin{array}{cc} {\left(\alpha_{E_{3}}\left(y\right),\alpha_{E_{3}}\left(z\right)\right)}_{s} & =\sum_{i=1}^{n}\left(\alpha_{E_{3}}\left(y_{i}\right)\alpha_{E_{3}}\left(z_{n+i}\right)-\alpha_{E_{3}}\left(y_{n+i}\right)\alpha_{E_{3}}\left(z_{i}\right)\right)\\ & =\alpha_{E_{3}}\left(\sum_{i=1}^{n}\left(y_{i}z_{n+i}-y_{n+i}z_{i}\right)\right)=\alpha_{E_{3}}\left({\left(y,z\right)}_{s}\right)\\ & =\alpha_{E_{3}}\left(0\right)=0. \end{array}$$

This derived that $\alpha_{E_{3}}\left(z\right)\in \mathrm{res}{\left(C\right)}^{{⊥}_{s}}$, which contradicts that res(C) is symplectic LCD. Hence, z=0. So, $C\cap C^{{⊥}_{R_{s}}}=\left\{0\right\}$.

Hence, *C* is a right-symplectic ACD code over $E_{3}$. □

The inverse statement of Corollary 3 is also true, as the corollary below establishes.

**Corollary** **4.**  *Let C be a free linear $E_{3}$ code with residue code is ternary symplectic LCD, then C is symplectic LCD.*

**Proof.**  Since res(C) is a symplectic LCD code, then to prove *C* is symplectic LCD is sufficient to check the conditions (2) and (3) of Proposition 7. We have *C* is an additive $E_{3}$ code, then by Theorem 5,

$$\begin{array}{cc} \mathrm{tor}\left(C\right)c\cap C^{{⊥}_{R_{s}}} & =\mathrm{tor}\left(C\right)c\cap {\langle \mathrm{res}{\left(C\right)}^{{⊥}_{s}}\rangle }_{E_{3}}=\mathrm{res}\left(C\right)c\cap {\langle \mathrm{res}{\left(C\right)}^{{⊥}_{s}}\rangle }_{E_{3}}\\ & =\mathrm{res}\left(C\right)c\cap \mathrm{res}{\left(C\right)}^{{⊥}_{s}}c=\left(\mathrm{res}\left(C\right)\cap \mathrm{res}{\left(C\right)}^{{⊥}_{s}}\right)c\\ & =\{0\}. \end{array}$$

This shows that condition (2) is satisfied. Therefore, from the freeness of *C*, we get $d_{1}=d_{2}$, so $3^{d_{1}+d_{2}}\times 9^{2n-d_{1}}=9^{d_{1}}\times 9^{2n-d_{1}}=9^{2n}$. This yields condition (3) is satisfied.

Hence, *C* is right-symplectic ACD. Moreover, *C* is a symplectic LCD code over $E_{3}$. □

We determine the residue and torsion codes of the right-symplectic dual codes of additive $E_{3}$ codes as the following theorem shows.

**Theorem** **6.**  *Let C be an additive code over $E_{3}$ of length 2n. Then,*

$$\mathrm{res}\left(C^{{⊥}_{R_{s}}}\right)=\mathrm{tor}\left(C^{{⊥}_{R_{s}}}\right)=\mathrm{res}{\left(C\right)}^{{⊥}_{s}}.$$

**Proof.**  By Lemma 3, $C^{{⊥}_{R_{s}}}$ is a free linear code, then $\mathrm{res}\left(C^{{⊥}_{R_{s}}}\right)=\mathrm{tor}\left(C^{{⊥}_{R_{s}}}\right)$. Then, we have to check that $\mathrm{res}\left(C^{{⊥}_{R_{s}}}\right)\subseteq \mathrm{res}{\left(C\right)}^{{⊥}_{s}}$. Let $xa+yc\in C^{{⊥}_{R_{s}}}$ and $x^{\prime }a+y^{\prime }c\in C$ where $x,x^{\prime },y,y^{\prime }\in F_{3}^{2n}$. Then, $\alpha_{E_{3}}\left(xa+yc\right)=x\in \mathrm{res}\left(C^{{⊥}_{R_{s}}}\right)$ and $\alpha_{E_{3}}\left(x^{\prime }a+y^{\prime }c\right)=x^{\prime }\in \mathrm{res}\left(C\right)$. Since $\alpha_{E_{3}}$ is a ring morphism and ${\left(x^{\prime }a+y^{\prime }c,xa+yc\right)}_{s}=0$, we get

$$\alpha_{E_{3}}\left({\left(x^{\prime }a+y^{\prime }c,xa+yc\right)}_{s}\right)={\left(\alpha_{E_{3}}\left(x^{\prime }a+y^{\prime }c\right),\alpha_{E_{3}}\left(xa+yc\right)\right)}_{s}={\left(x^{\prime },x\right)}_{s}=0.$$

This implies that $x\in \mathrm{res}{\left(C\right)}^{{⊥}_{s}}$. So, $\mathrm{res}\left(C^{{⊥}_{R_{s}}}\right)\subseteq \mathrm{res}{\left(C\right)}^{{⊥}_{s}}$.

To prove that $\mathrm{res}{\left(C\right)}^{{⊥}_{s}}\subseteq \mathrm{res}\left(C^{{⊥}_{R_{s}}}\right)$, suppose x is an arbitrary element of $\mathrm{res}{\left(C\right)}^{{⊥}_{s}}$. Let $z=ya+y^{\prime }c\in C$ where $y,y^{\prime }\in F_{3}^{2n}$. Then, $\alpha_{E_{3}}\left(z\right)=y\in \mathrm{res}\left(C\right)$. So,

$${\left(z,xa\right)}_{s}={\left(ya+y^{\prime }c,xa\right)}_{s}={\left(ya,xa\right)}_{s}+{\left(y^{\prime }c,xa\right)}_{s}={\left(ya,xa\right)}_{s}={\left(y,x\right)}_{s}a+0=0.$$

This shows that $xa\in C^{{⊥}_{R_{s}}}$. Thus, $x\in \mathrm{res}(C^{{⊥}_{R_{s}}})$ as $\alpha_{E_{3}}\left(xa\right)=x$. Hence, $\mathrm{res}{\left(C\right)}^{{⊥}_{s}}\subseteq \mathrm{res}\left(C^{{⊥}_{R_{s}}}\right)$. Therefore, $\mathrm{res}\left(C^{{⊥}_{R_{s}}}\right)=\mathrm{res}{\left(C\right)}^{{⊥}_{s}}$. □

**Corollary** **5.**  *For any linear $E_{3}$ code, C, we have $\mathrm{tor}{\left(C\right)}^{{⊥}_{s}}\subseteq \mathrm{tor}\left(C^{{⊥}_{R_{s}}}\right)$. Particularly, if C is free, then $\mathrm{tor}\left(C^{{⊥}_{R_{s}}}\right)=\mathrm{tor}{\left(C\right)}^{{⊥}_{s}}$.*

**Proof.**  First we have, res(C)⊆tor(C) as *C* is a linear $E_{3}$ code, then $\mathrm{tor}{\left(C\right)}^{{⊥}_{s}}\subseteq \mathrm{res}{\left(C\right)}^{{⊥}_{s}}$. By Theorem 6, $\mathrm{res}\left(C^{{⊥}_{R_{s}}}\right)=\mathrm{res}{\left(C\right)}^{{⊥}_{s}}⊇\mathrm{tor}{\left(C\right)}^{{⊥}_{s}}$. According to Lemma 3, $C^{{⊥}_{R_{s}}}$ is free, so $\mathrm{tor}\left(C^{{⊥}_{R_{s}}}\right)=\mathrm{res}\left(C^{{⊥}_{R_{s}}}\right)$. Hence, $\mathrm{tor}{\left(C\right)}^{{⊥}_{s}}\subseteq \mathrm{tor}\left(C^{{⊥}_{R_{s}}}\right)$. Therefore, if *C* is free, then tor(C)=res(C). Thus, $\mathrm{tor}{\left(C\right)}^{{⊥}_{s}}=\mathrm{res}{\left(C\right)}^{{⊥}_{s}}$. By Lemma 3 and Theorem 6, $\mathrm{tor}\left(C^{{⊥}_{R_{s}}}\right)=\mathrm{res}\left(C^{{⊥}_{R_{s}}}\right)=\mathrm{res}{\left(C\right)}^{{⊥}_{s}}$. Hence, $\mathrm{tor}\left(C^{{⊥}_{R_{s}}}\right)=\mathrm{tor}{\left(C\right)}^{{⊥}_{s}}$. □

The converse statement of Proposition 7 partially holds.

**Proposition** **8.**  *For an additive $E_{3}$ code, C, of length 2n. We have, $\mathrm{tor}\left(C\right)c\cap C^{{⊥}_{R_{s}}}=\left\{0\right\}$, res(C) is a ternary symplectic LCD code and $3^{d_{1}+d_{2}}\times 9^{2n-d_{1}}=9^{2n}$ (i.e., $d_{1}=d_{2}$) if C is a right-symplectic ACD code over $E_{3}$ with $Cc\cap C^{{⊥}_{R_{s}}}=\left\{0\right\}$.*

**Proof.**  Let $z\in \mathrm{tor}\left(C\right)c\cap C^{{⊥}_{R_{s}}}$. Then, z∈tor(C)c; this means z=tc with t∈tor(C). By the definition of the torsion code, z∈C. This together with $z\in C^{{⊥}_{R_{s}}}$ imply that $z\in C\cap C^{{⊥}_{R_{s}}}$. Since *C* is right-symplectic ACD code, z=0. Hence, $\mathrm{tor}\left(C\right)c\cap C^{{⊥}_{R_{s}}}=\left\{0\right\}$.

We have $\left|C\right|=\left|\mathrm{res}\left(C\right)||\mathrm{tor}\left(C\right)\right|=3^{d_{1}+d_{2}}$ and by Theorem 5, $|C^{{⊥}_{R_{s}}}|\;=9^{2n-d_{1}}$. Therefore, $\left|C|\right|C^{{⊥}_{R_{s}}}|\;=3^{d_{1}+d_{2}}\times 9^{2n-d_{1}}$. Since *C* is right-symplectic-nice, $3^{d_{1}+d_{2}}\times 9^{2n-d_{1}}=9^{2n}$. Hence, $d_{1}=d_{2}$. Now, let $r\in \mathrm{res}\left(C\right)\cap \mathrm{res}{\left(C\right)}^{{⊥}_{s}}$. By Theorem 6, $r\in \mathrm{res}{\left(C\right)}^{{⊥}_{s}}=\mathrm{res}\left(C^{{⊥}_{R_{s}}}\right)$. Thus, there are ra+ta∈C and $ra+t^{\prime }a\in C^{{⊥}_{R_{s}}}$ such that $\alpha_{E_{3}}\left(ra+ta\right)=\alpha_{E_{3}}\left(ra+t^{\prime }a\right)=r$. From the linearity of $C^{{⊥}_{R_{s}}}$ over $E_{3}$, we get $\left(ra+t^{\prime }a\right)c=rc\in C^{{⊥}_{R_{s}}}$. Therefore, $(ra+t^{\prime }a)c=rc\in Cc$. So, $\left(ra+t^{\prime }a\right)c\in Cc\cap C^{{⊥}_{R_{s}}}=\left\{0\right\}$. Thus, rc=0, also r=0. Hence, res(C) is a ternary symplectic LCD code. □

**Proposition** **9.**  *Let C be an additive code of length 2n over $E_{3}$. Then, $\mathrm{tor}\left(C^{{⊥}_{L_{s}}}\right)\subseteq \mathrm{res}{\left(C\right)}^{{⊥}_{s}}$. Equality holds if C is linear.*

**Proof.**  Let $z=ra+tc\in C^{{⊥}_{L_{s}}}$ where $r,t\in F_{3}^{2n}$. Then, $\alpha_{E_{3}}\left(z\right)=\alpha_{E_{3}}\left(ra+tc\right)=r$ is an arbitrary codeword of $\mathrm{res}(C^{{⊥}_{L_{s}}})$. Therefore, let $t^{\prime }\in \mathrm{tor}\left(C\right)$, then $t^{\prime }c\in C$. This together with $z\in C^{{⊥}_{L_{s}}}$, we get

$$0={\left(z,t^{\prime }c\right)}_{s}={\left(ra+tc,t^{\prime }c\right)}_{s}={\left(ra,t^{\prime }c\right)}_{s}+{\left(tc,t^{\prime }c\right)}_{s}={\left(r,t^{\prime }\right)}_{s}c.$$

This yields ${\left(r,t^{\prime }\right)}_{s}=0$. Hence, $r\in \mathrm{tor}(C^{{⊥}_{L_{s}}})$. So, $\mathrm{tor}\left(C^{{⊥}_{L_{s}}}\right)\subseteq \mathrm{res}{\left(C\right)}^{{⊥}_{s}}$.

Now, suppose that *C* is a linear code. Then, let z∈C. By Theorem 3.5 in [8], z=xa+yc for some x∈res(C) and y∈tor(C). Let $y^{\prime }\in \mathrm{tor}{\left(C\right)}^{{⊥}_{s}}$, we have res(C)⊆tor(C). Then, we obtain

$${\left(y^{\prime }a,z\right)}_{s}={\left(y^{\prime }a,y^{\prime }a+yc\right)}_{s}={\left(y^{\prime }a,xa\right)}_{s}+{\left(y^{\prime }a,yc\right)}_{s}={\left(y^{\prime },x\right)}_{s}a+{\left(y^{\prime },y\right)}_{s}c=0+0=0.$$

Hence, $y^{\prime }a\in C^{{⊥}_{L_{s}}}$. This implies that, $\alpha_{E_{3}}\left(y^{\prime }a\right)=y^{\prime }\in \mathrm{res}\left(C^{{⊥}_{L_{s}}}\right)$. So, $\mathrm{tor}{\left(C\right)}^{{⊥}_{s}}\subseteq \mathrm{res}\left(C^{{⊥}_{L_{s}}}\right)$. Therefore, $\mathrm{res}\left(C^{{⊥}_{L_{s}}}\right)=\mathrm{tor}{\left(C\right)}^{{⊥}_{s}}$. □

**Proposition** **10.**  *For a linear $E_{3}$ code, C, of length 2n. We have,*

$$C\mathrm{is}\mathrm{free}\mathrm{if},\;\mathrm{and}\mathrm{only}\mathrm{if},\;\mathrm{res}\left(C^{{⊥}_{R_{s}}}\right)=\mathrm{res}\left(C^{{⊥}_{L_{s}}}\right)$$

**Proof.**  Suppose *C* is a linear $E_{3}$ code. By Theorem 6, $\mathrm{res}\left(C^{{⊥}_{R_{s}}}\right)=\mathrm{res}{\left(C\right)}^{{⊥}_{s}})$. According to Proposition 9, $\mathrm{res}\left(C^{{⊥}_{L_{s}}}\right)=\mathrm{tor}{\left(C\right)}^{{⊥}_{s}}$. Since *C* is free, res(C)=tor(C). Thus, $\mathrm{res}{\left(C\right)}^{{⊥}_{s}}=\mathrm{tor}{\left(C\right)}^{{⊥}_{s}}$. Hence, $\mathrm{res}\left(C^{{⊥}_{R_{s}}}\right)=\mathrm{res}\left(C^{{⊥}_{L_{s}}}\right)$.

For the inverse assertion. Assume, *C* is a linear $E_{3}$ code with $\mathrm{res}\left(C^{{⊥}_{R_{s}}}\right)=\mathrm{res}\left(C^{{⊥}_{L_{s}}}\right)$. By Theorem 6 and Proposition 9, $\mathrm{tor}{\left(C\right)}^{{⊥}_{s}}=\mathrm{res}{\left(C\right)}^{{⊥}_{s}}$. So, tor(C)=res(C). Hence, *C* is free. □

In what follows, we give an example to describe Theorem 4 and Proposition 7.

**Example** **2.**  *Let C be an additive $E_{3}$ code of length *4* and additive generator matrix*

$$G=\left[\begin{array}{cccc} a & 2b & 0 & 0\\ 0 & 0 & a & 2b \end{array}\right]$$

*Let $\left(y,y^{\prime },z,z^{\prime }\right)\in C^{{⊥}_{R_{s}}}$. Then,*

$${\left(\left(a,2b,0,0\right),\left(y,y^{\prime },z,z^{\prime }\right)\right)}_{s}=0\;and\;{\left(\left(0,0,a,2b\right),\left(y,y^{\prime },z,z^{\prime }\right)\right)}_{s}=0.$$

*These imply that $az+2bz^{\prime }=0$ and $2ay+by^{\prime }=0$. Since ax=bx=x for any $x\in E_{3}$, we get $z+2z^{\prime }=0$ and $2y+y^{\prime }=0$. So, $z^{\prime }=z$ and $y^{\prime }=y$. Hence, $C^{{⊥}_{R_{s}}}=\left\{\left(y,y,z,z\right)|\;y,z\in E_{3}\right\}$.*

*It is easy to derive, $C\cap C^{{⊥}_{R_{s}}}=Cc\cap C^{{⊥}_{R_{s}}}=\left\{0\right\}$. Furthermore, |C| =9 and $|C^{{⊥}_{R_{s}}}|\;=9^{2}$. So, $\left|C|\right|C^{{⊥}_{R_{s}}}|\;=9^{3}<9^{4}$. We add (a,2b,0,0)c=(c,2c,0,0) and (0,0,a,2b)c=(0,0,2c,c) to G, then we get $G^{\prime }$ where*

$$G^{\prime }=\left[\begin{array}{cccc} a & 2b & 0 & 0\\ 0 & 0 & a & 2b\\ c & 2c & 0 & 0\\ 0 & 0 & c & 2c \end{array}\right]$$

*Let $C^{\prime }$ be the additive $E_{3}$–code of length *4* with an additive generator matrix $G^{\prime }$. Then, according to Theorem 4, $C^{\prime }$ is right-symplectic ACD over $E_{3}$.*

*To prove $C^{\prime }$ is right-symplectic ACD, we check is this code satisfies the conditions of Proposition 7. First, we prove that $\mathrm{res}(C^{\prime })$ is ternary symplectic LCD code. Let $\left(z_{1},z_{2},z_{3},z_{4}\right)\in \mathrm{res}{\left(C^{\prime }\right)}^{{⊥}_{s}}$. Then,*

$${\left(\left(y,y^{\prime },z,z^{\prime }\right),\left(1,2,0,0\right)\right)}_{s}=0\;and\;{\left(\left(y,y^{\prime },z,z^{\prime }\right),\left(0,0,1,2\right)\right)}_{s}=0.$$

*Thus, $2z+z^{\prime }=0$ and $2y+y^{\prime }=0$. So, $z^{\prime }=z_{3}$ and $y^{\prime }=y$. Hence, $\mathrm{res}{\left(C^{\prime }\right)}^{{⊥}_{s}}=\left\{\left(y,y,z,z\right)|;\;z\in F_{3}\right\}$. This gives $\mathrm{res}\left(C^{\prime }\right)\cap \mathrm{res}{\left(C^{\prime }\right)}^{{⊥}_{s}}=\left\{0\right\}$. Hence, $\mathrm{res}(C^{\prime })$ is a ternary symplectic LCD.*

*Clearly, $\mathrm{res}(C^{\prime })$ has the generator matrix $G_{1}^{\prime }=\left[\begin{array}{cccc} 1 & 2 & 0 & 0\\ 0 & 0 & 1 & 2 \end{array}\right]$ and the generator matrix of $\mathrm{tor}(C^{\prime })$ is $G_{2}^{\prime }=\left[\begin{array}{cccc} 1 & 2 & 0 & 0\\ 0 & 0 & 1 & 2 \end{array}\right]$. These show that $d_{1}=d_{2}=2$.*

*Therefore, $\mathrm{tor}(C^{\prime })c$ has generator matrix $\left[\begin{array}{cccc} c & 2c & 0 & 0\\ 0 & 0 & c & 2c \end{array}\right]$.*

*By Theorem 5, $C^{\prime {⊥}_{R_{s}}}={\langle \mathrm{res}{\left(C^{\prime }\right)}^{{⊥}_{s}}\rangle }_{E_{3}}$. It is easy to see that, $\mathrm{tor}\left(C^{\prime }\right)c\cap C^{\prime {⊥}_{R_{s}}}=\left\{0\right\}$.*

*Hence, $C^{\prime }$ is right-symplectic ACD code over $E_{3}$ according to Proposition 7.*

## 5. Conclusions

We have introduced symplectic QSD, LCD, and ACD codes over non-commutative non-unitary local ring $E_{3}$ with nine elements. Using symplectic–self-orthogonal ternary linear codes, we have given the multi-level construction of symplectic QSD codes. We have shown the relationship between symplectic LCD $E_{3}$ codes and ternary symplectic LCD codes. Further, the right-symplectic ACD $E_{3}$ codes were introduced and for the existence of such codes, and several conditions have been stated. We have also characterized the right-symplectic ACD codes over $E_{3}$.

The construction of quantum codes can be achieved by deriving classical codes over finite fields with the symplectic–self-orthogonal property [14,15]. Therefore, these codes could be applied to construct quantum codes over this ring.

Additionally, it will be interesting to derive a mass formula for symplectic QSD and self-dual $E_{3}$ codes and classify them in modest lengths. Moreover, the absence of non-trivial symplectic left LCD codes over $E_{3}$ makes the study of symplectic left hull codes over this ring a natural direction for future work.

## Figures and Tables

**Table 1:** Multiplication table for $E_{3}$.

×	0	*a*	*b*	*c*	2a	2b	2c	a+b	a+c
0	0	0	0	0	0	0	0	0	0
*a*	0	*a*	*b*	*c*	2a	2b	2c	a+b	a+c
*b*	0	*a*	*b*	*c*	2a	2b	2c	a+b	a+c
*c*	0	0	0	0	0	0	0	0	0
2a	0	2a	2b	2c	*a*	*b*	*c*	a+c	a+b
2b	0	2a	2b	2c	*a*	*b*	*c*	a+c	a+b
2c	0	0	0	0	0	0	0	0	0
a+b	0	2a	2b	2c	*a*	*b*	*c*	a+c	a+b
a+c	0	*a*	*b*	*c*	2a	2b	2c	a+b	a+c

## Data Availability

The raw data supporting the conclusions of this article will be made available by the authors on request.
